# Phenotype Frequencies of Autosomal Minor Histocompatibility Antigens Display Significant Differences among Populations

**DOI:** 10.1371/journal.pgen.0030103

**Published:** 2007-06-29

**Authors:** Eric Spierings, Matthijs Hendriks, Léna Absi, Angelica Canossi, Sonal Chhaya, John Crowley, Harry Dolstra, Jean-François Eliaou, Tom Ellis, Jürgen Enczmann, Maria E Fasano, Thibaut Gervais, Clara Gorodezky, Brigitte Kircher, David Laurin, Mary S Leffell, Pascale Loiseau, Mari Malkki, Miroslaw Markiewicz, Miryam Martinetti, Etsuko Maruya, Narinder Mehra, Fatma Oguz, Machteld Oudshoorn, Noemi Pereira, Rajni Rani, Ruhena Sergeant, Jackie Thomson, Thuong Hien Tran, Hannu Turpeinen, Kuo-Liang Yang, Renata Zunec, Mary Carrington, Peter de Knijff, Els Goulmy

**Affiliations:** 1 Immunohematology and Blood Transfusion, Leiden University Medical Center, Leiden, The Netherlands; 2 Laboratoire d'Histocompatibilité, EFS Auvergne Loire, St Etienne, France; 3 Istituto CNR Trapianti D'Organo e l'Immunocitologia, L'Aquila, Italy; 4 Transfusion Medicine, Tata Memorial Hospital, Mumbai, India; 5 National Histocompatibility and Immunogenetics Reference Laboratory, National Blood Centre, Dublin, Ireland; 6 Central Hematology Laboratory, Radboud University Nijmegen Medical Centre, Nijmegen, The Netherlands; 7 CHU Montpellier Unité d'Immunogénétique, Laboratoire d'Immunologie, Hopital Saint-Eloi, Montpellier, France; 8 Histocompatibility and Immunogenetics, Blood Center of Southeastern Wisconsin, Milwaukee, Wisconsin, United States of America; 9 Institute for Transplantation Immunology, University Hospital, Düsseldorf, Germany; 10 Immunologia dei Trapianti, Az. Ospedaliera S.Giovanni Battista di Torino U.O.A.D.U., Torino, Italy; 11 Immunohaematology, Cliniques St Luc, Université Catholique de Louvain, Brussels, Belgium; 12 Department of Immunology and Immunogenetics, Instituto de Diagnostico y Referencia Epidemiologicos, México City, Mexico; 13 Immunobiology and Stem Cell Laboratory, Division of Hematology and Oncology, Innsbruck Medical University, Innsbruck, Austria; 14 Laboratoire d'Immunologie Research and Development, Establissement Francais du Sang Rhône-Alpes, Grenoble, France; 15 Immunogenetics Laboratory, Johns Hopkins University School of Medicine, Baltimore, Maryland, United States of America; 16 Laboratoire d'Immunologie, Hopital Saint-Louis, Paris, France; 17 Division of Clinical Research, Fred Hutchinson Cancer Research Center, Seattle, Washington, United States of America; 18 Hematology and BMT Department, Medical University of Silesia, Katowice, Poland; 19 Laboratorio HLA, Servizio di Immunoematologia e Trasfusione, IRCCS Policlinico S.Matteo, Pavia, Italy; 20 HLA Laboratory, Kawabatadohri-marutamachi, Sakyo-ku, Kyoto, Japan; 21 Department of Transplant Immunology and Immunogenetics, All India Institute of Medical Sciences, New Delhi, India; 22 Department of Medical Biology, Medical Faculty of Istanbul, Istanbul University, Istanbul, Turkey; 23 Stichting Europdonor, Leiden, The Netherlands; 24 Laboratorio de Imunogenetica, Universidade Federal do Parana Hospital de Clinicas, Curitiba, Brasil; 25 National Institute of Immunology, New Delhi, India; 26 Clinical Immunology Laboratory, Hammersmith Hospital, London, United Kingdom; 27 Laboratory for Tissue Immunology, University of Cape Town Medical School, Cape Town, South Africa; 28 Department of Transplantation Immunology, Institute of Immunology, University of Heidelberg, Heidelberg, Germany; 29 Research and Development, Finnish Red Cross Blood Service, Helsinki, Finland; 30 Cord Blood Bank, Tzu Chi Stem Cells Centre, Hualien, Taiwan; 31 Tissue Typing Centre, Clinical Hospital Zagreb, Zagreb, Croatia; 32 Laboratory of Genomic Diversity, Science Applications International Corporation-Frederick, Frederick, Maryland, United States of America; 33 National Cancer Institute–Frederick Cancer Research and Development Center, Frederick, Maryland, United States of America; 34 Department of Human Genetics, Leiden University Medical Center, Leiden, The Netherlands; The Jackson Laboratory, United States of America

## Abstract

Minor histocompatibility (H) antigens are allogeneic target molecules having significant roles in alloimmune responses after human leukocyte antigen–matched solid organ and stem cell transplantation (SCT). Minor H antigens are instrumental in the processes of transplant rejection, graft-versus-host disease, and in the curative graft-versus-tumor effect of SCT. The latter characteristic enabled the current application of selected minor H antigens in clinical immunotherapeutic SCT protocols. No information exists on the global phenotypic distribution of the currently identified minor H antigens. Therefore, an estimation of their overall impact in human leukocyte antigen–matched solid organ and SCT in the major ethnic populations is still lacking. For the first time, a worldwide phenotype frequency analysis of ten autosomal minor H antigens was executed by 31 laboratories and comprised 2,685 randomly selected individuals from six major ethnic populations. Significant differences in minor H antigen frequencies were observed between the ethnic populations, some of which appeared to be geographically correlated.

## Author Summary

Peptides from cellular proteins evoking alloimmune responses in human leukocyte antigen–identical transplantation are called minor histocompatibility (H) antigens. Upon hematopoietic stem cell transplantation (HSCT) for hematological malignancies or for solid tumors, responses to minor H antigens may have detrimental effects, e.g., graft-versus-host-disease and graft rejection, but can also significantly contribute to the eradication of the tumor cells. We designated the latter antigens as “tumor” minor H antigens. Current clinical trials aim at using these tumor minor H antigens to boost the graft-versus-tumor response. So far, it is unclear how frequently the HSCT recipient and donor differ in their minor H antigens and thus how many cancer patients are eligible for minor H antigen-based treatment. Therefore, worldwide 31 laboratories joined forces to determine the genotype and phenotype frequencies of ten autosomally encoded minor H antigens in six ethnic populations. The frequencies vary depending upon ethnic background and geographic location of the population, implying that the potential applicability of the tumor minor H antigens differs from one population to another. Depending on the population, the current theoretical percentages of clinical application of the eight tumor minor H antigens in HLA-matched combinations is estimated at 12% to 34%.

## Introduction

Minor histocompatibility (H) antigens are polymorphic peptides that are presented on the cell surface by major histocompatibility complex class I or II molecules [1]. These peptides are derived from allelic cellular proteins encoded by autosomal genes or by genes of the Y chromosome [2]. In general, minor H antigen-encoding proteins are biallelic, encoding an immunogenic and a nonimmunogenic allele. Individuals homozygous for the nonimmunogenic minor H allele can develop immune responses to cells expressing the immunogenic minor H allele. Minor H antigen alloimmune responses readily occur in the setting of human leukocyte antigen (HLA)–matched allogeneic solid organ and stem cell transplantation (SCT) [3,4]. The role of minor H antigen disparities is most thoroughly analyzed in the setting of HLA–matched SCT, wherein powerful minor H antigen alloimmune reactivities have been documented in the graft-versus-host direction (for review see [5]). Minor H antigen responses are detected during the development of graft-versus-host disease (GvHD) and during the curative graft-versus-tumor (GvT) responses after HLA-matched SCT (for review see [6]). This seemingly conflicting role of the minor H antigens in the two GvH (i.e., GvHD and GvT reaction) responses was dissected by the differential tissue distribution of minor H antigens. In vitro studies showed differential modes of expression of the various minor H antigens being either ubiquitously expressed or with expression restricted to the hematopoietic system [7,8]. Ubiquitously expressed minor H antigens are present on many cells and tissues, including the GvHD target organs: skin, liver, and gut. These minor H antigens are therefore particularly relevant in the development of GvHD. Hematopoietic system–restricted minor H antigens are only expressed by hematopoietic cells and their progenitors with no expression on nonhematopoietic cells. Some of these minor H antigens can also be expressed by tumor cells [9–12]. Minor H antigens with tissue expression limited to cells of the hematopoietic system and/or tumor cells are especially relevant for the GvT activity.

For example, cytotoxic T cells specific to the hematopoietic-restricted minor H antigens HA-1 and HA-2 eradicate circulating leukemic cells and leukemic progenitors cells in vitro [13,14] and in an in vivo translational mouse model [15] and can be isolated in the course of remission of donor lymphocyte infusion–treated patients after HLA-matched SCT [16,17]. Currently, hematopoietic system- and tumor-specific minor H antigenic peptides are being applied in clinical phase I and II vaccination studies to boost the GvT response (reviewed in [6]).

Although scarcely analyzed, minor H antigens are likely to function as potential risks for graft rejection of HLA-matched solid organ transplants [18]. The apparent need of the organ transplant recipient for lifelong immunosuppressive drugs supports this notion. Interestingly, a beneficial effect of minor H antigen mismatching on long-term HLA-matched renal allograft survival has been recently described [19]. Namely, HA-1 specific T regulator cells were present in a patient who had discontinued immune suppression over 30 y ago while sustaining normal kidney function. Herewith a novel characteristic for human minor H antigens has been disclosed with potential impact for decrement of immunosuppressive therapy in solid organ grafting.

Minor H antigen alloimmune responses also occur in the physiological setting of pregnancy. Pregnancy leads to a mutual flow of cells between mother and child. The exposure to either foreign maternal or paternal minor H antigens during pregnancy drives the generation of minor H antigen specific T cells in mutual direction [20,21]. To date, it is clear that minor H antigenic responses cannot be ignored in the nonphysiological situation of transplantation and in the physiological setting of pregnancy, where female donors with a history of pregnancy and cord blood are used as sources for SCT. Moreover, various minor H antigen identification systems recently became available, facilitating their molecular identification (for overview see [2]). It is therefore timely to estimate the potential impact of the minor H antigens molecularly identified today in the different settings described above. Furthermore, since the first minor H antigen–based clinical vaccination trials in SCT protocols have begun, it is of great importance to estimate their potential applicability in different ethnic groups. Hereto, information on the phenotype frequencies of minor H antigens is required. Population frequency analyses of minor H antigens have so far been performed for a limited number of minor H antigens and are mainly limited to the White population [22]. Additional but scarce information on some of the minor H antigen genotypes is provided by the HapMap project [23]. The present multicenter study reports on a worldwide analysis providing for the first time the global phenotype frequencies of the ten autosomally encoded minor H antigens identified at the initiation of the study. Using these data, geographic patterns were additionally analyzed. Moreover, the theoretical clinical usefulness in HLA-matched SCT of each individual minor H antigen was estimated within six different ethnic populations.

## Results

### Study Populations

Genomic typing data of ten autosomally encoded minor H antigens were obtained from 2,685 individuals from five different continents comprising 16 different countries and five different ethnic groups. The backgrounds of the five ethnic groups were: (1) 305 Asian/Pacific Islanders, 11.4%; (2) 162 Black, 6.0%; (3) 2,011 White, 74.9%; (4) 119 Mestizo Mexican, 4.4%; and (5) 88 specified as “others,” 3.3%. The Asian/Pacific Islander group could be subdivided into Southeast Asian/Southern Chinese (10.8% of the Asian/Pacific Islander group), Asian Indian (67.9%), Japanese (20.3%), and Asian not otherwise specified (1.0%). The Black population consisted of 102 African Americans (63.0% of the Black population), 54 African Black both parents born in Africa (33.2%), three South or Central American Black (1.9%), and three were Black not otherwise specified (1.9%). The White population originated from Europe (82.1%), Australia (0.6%), South Africa (0.5%), Southern America (6.1%), and Northern America (10.7%). The group “others” comprised a major population of Cape Colored individuals from South Africa (*n* = 65; 2.4% of the study population) and a Mulatto population from Brazil (*n* = 23; 0.9%). Both groups comprise individuals of mixed ethnicity.

### Minor H Antigen Phenotype Frequencies

#### HA-1*.*

The HLA-A2-restricted immunogenic HA-1 phenotype (i.e., HA-1^HH^ and HA-1^HR^ genotypes; see Table 1 for a complete list of the immunogenic and nonimmunogenic minor H alleles and Table 2 for their allele frequencies) is most abundant in the Black population (73.7%; Table 3 and Table S1), followed by the Asian/Pacific population (71.2%) and the Mexican Mestizos (69.6%). In the White and the Cape Colored populations the frequency is substantially lower (58.8% and 61.4%, respectively). The differences between the White population compared to the Black, Asian/Pacific, or Mexican Mestizo populations reached statistical significance (*p* < 0.001, *p* < 0.001, and *p* < 0.025, respectively).

**Table 1:**
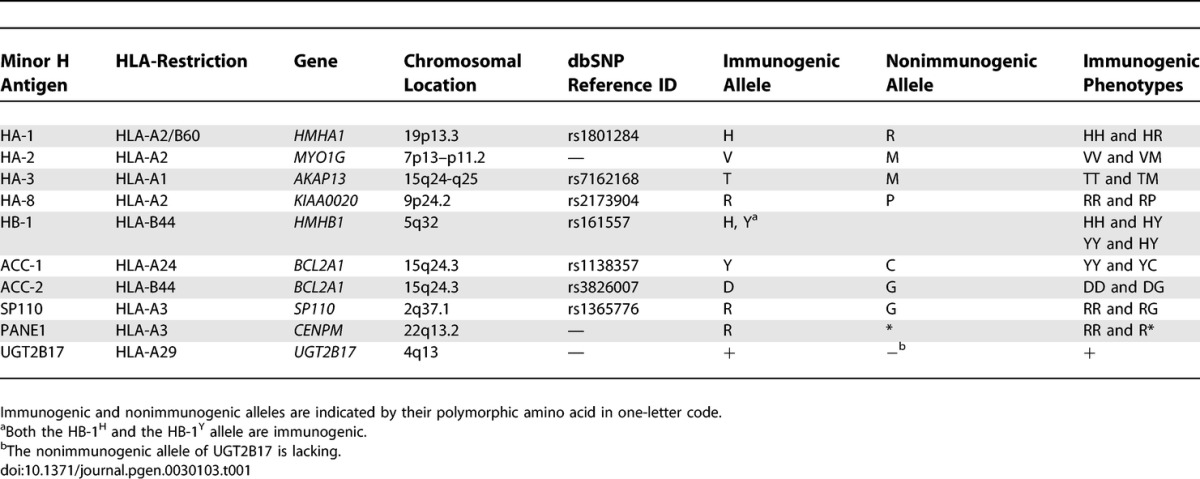
Overview of the Characteristics of Ten Minor H Antigens Investigated in this Study

**Table 2:**
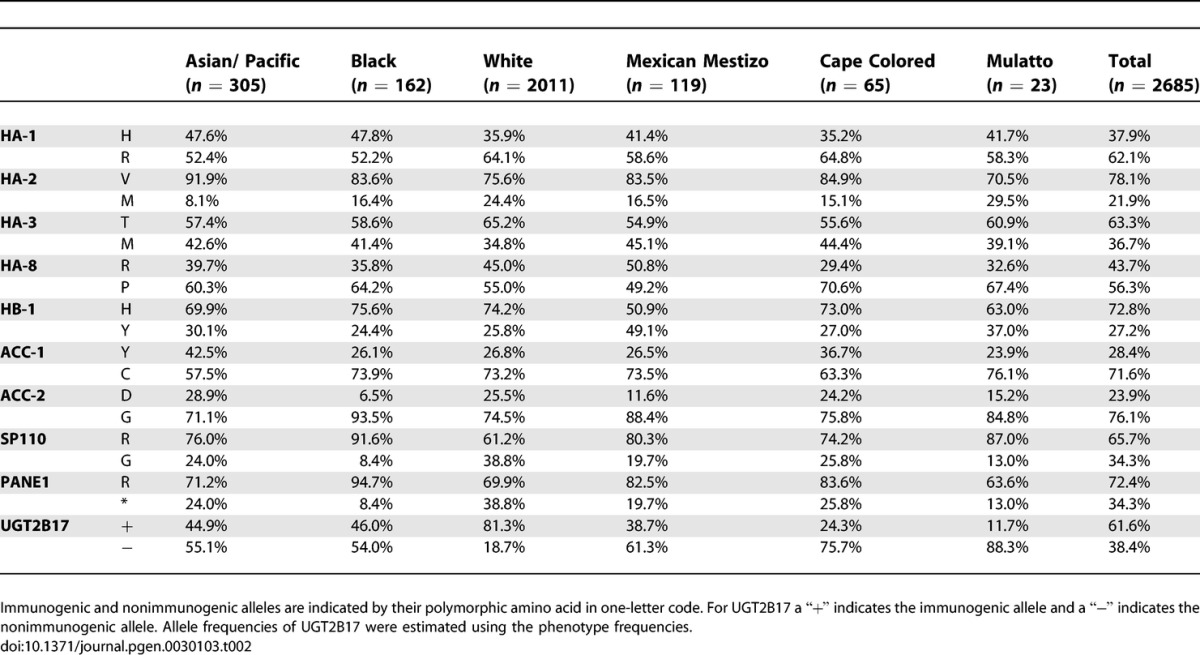
Frequencies of the Alleles of Ten Autosomally Encoded Minor H Antigens in Six Different Ethnic Populations

**Table 3:**
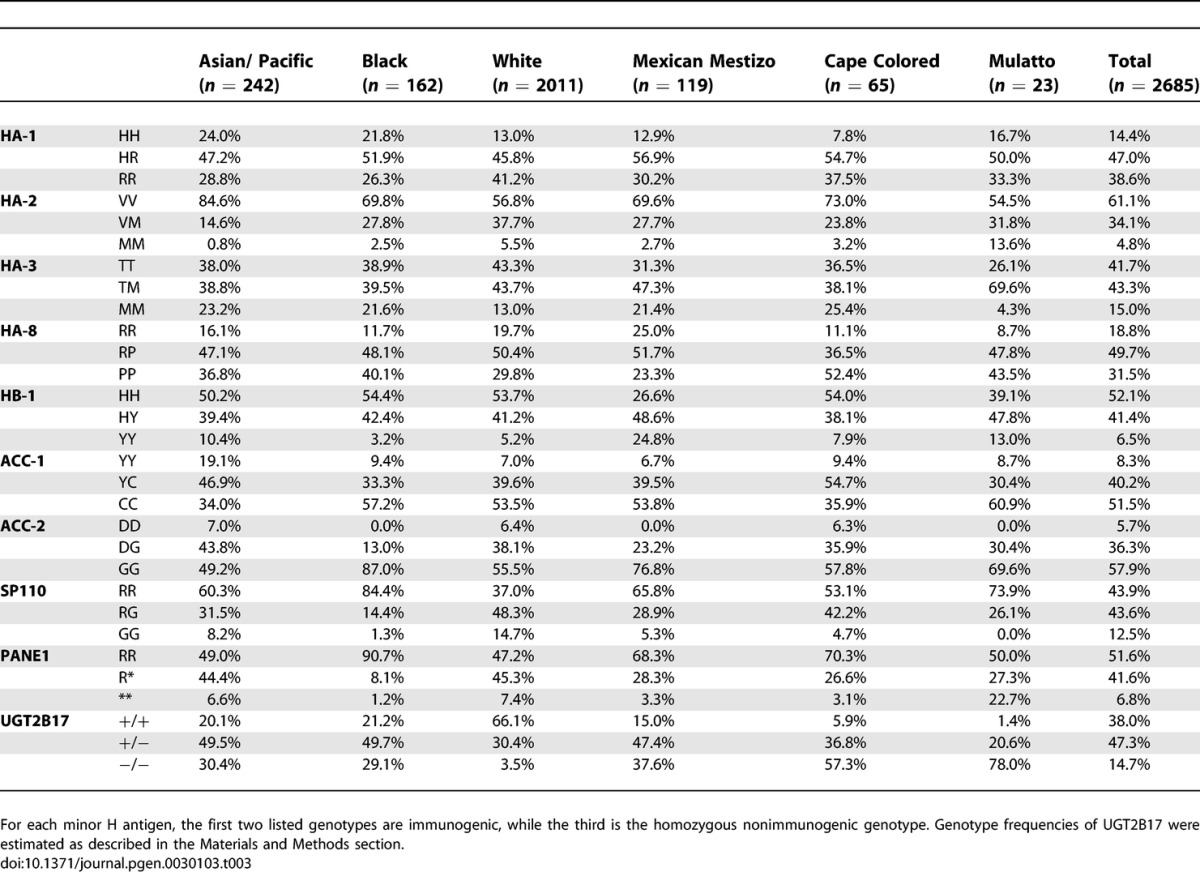
Genotype Frequencies of Ten Autosomally Encoded Minor H Antigens in Six Different Ethnic Populations

#### HA-2.

The HLA-A2-restricted immunogenic HA-2 phenotype frequency (i.e., HA-2^VV^ and HA-2^VM^ genotypes) has an average of 95.2% and is equally high in all populations studied (Table 3). The Brazilian Mulatto population has a frequency (86.3%) significantly lower than the Asian/Pacific (99.2%; *p* < 0.001), Black (97.5%; *p* < 0.025), and Mexican Mestizo populations (97.3%; *p* < 0.025).

#### HA-3.

The HLA-A1-restricted immunogenic HA-3 phenotype (i.e., HA-3^TT^ and HA-3^TM^) has a high frequency in the White (87.0%) and Mulatto populations (95.7%). The frequency of HA-3 is significantly lower (approximately 75%) in all other populations (White versus rest, *p* < 0.001; Mulatto versus rest, *p* = 0.05).

#### HA-8.

The frequency of the HLA-A2-restricted immunogenic HA-8 phenotype (i.e., HA-8^RR^ and HA-8^RP^) shows a broad range of frequencies with 47.6% in the Cape Colored population and 76.7% in the Mexican Mestizos.

#### HB-1.

For HB-1, two immunogenic alleles (HB-1^H^ and HB-1^Y^) presented by the HLA-B44 molecule have been described [24]. Phenotypes frequencies were therefore calculated for both the HB-1^H^ and the HB-1^Y^ T-cell epitopes (see Table S1). The frequency of the immunogenic HB-1^H^ phenotype (i.e., HB-1^HH^ and HB-1^HY^ genotypes) is 94.8% in the White population, 96.8% in the Black population, 92.1% in the Cape Colored population, and 87.0% in the Brazilian Mulatto population. The differences between these populations are not statistically significant. When compared to the first three populations, the HB-1^H^ phenotype is significantly different in the Asian/Pacific (89.6%, *p* < 0.001) and in the Mexican Mestizo (75%, *p* < 0.001) populations. The HLA-B44-restricted immunogenic HB-1^Y^ allele (i.e., HB-1^HY^ and HB-1^YY^ genotypes) occurs in approximately 46% of the individuals in the Asian/Pacific, Black, White, and Cape Colored populations. This phenotype is observed at a higher frequency in the Mexican Mestizo (73.4%, *p* < 0.001) and the Mulatto (60.9%, not significant) populations.

#### ACC-1.

The HLA-A24-restricted ACC-1 immunogenic phenotype (i.e., ACC-1^YY^ and ACC-1^YC^ genotypes) is at average observed in 48.5% of the analyzed individuals. The Asian/Pacific and the Cape Colored population have significantly higher frequencies with 66.0% (*p <* 0.001) and 64.1% (*p <* 0.01), respectively, more than the Black, White, Mexican Mestizo, and Mulatto populations.

#### ACC-2.

The HLA-B44-restricted ACC-2 immunogenic phenotype (i.e., ACC-1^DD^ and ACC-1^DG^ genotypes) frequency shows a diverse distribution. In the Asian/Pacific, White, and Cape Colored population, the immunogenic phenotype has a frequency of approximately 45%, while this frequency is only 30.4% in the Brazilian Mulattos (not significant), 13.0% in the Black population (*p <* 0.001), and 23.2% in the Mexican Mestizo population (not significant).

#### SP110.

The HLA-A3-restricted SP110 immunogenic phenotype (i.e., SP110^RR^ and SP110^RG^ genotypes) displays a very high frequency in the non-White populations (90%–100%), which is significantly lower in the White population (85.3%, *p <* 0.001).

#### PANE1.

The HLA-A3-restricted PANE1 immunogenic phenotype (i.e., PANE1^RR^ and PANE1^R*^ genotypes) has frequencies in almost all populations of 90%–100%. The most significant deviation was observed in the Mulatto population with 77.3% (*p <* 0.01).

#### UGT2B17.

The gene encoding UGT2B17 has only one functional allele; its allelic counterpart is deleted. Therefore, the immunogenic phenotype is defined as the presence of the gene on at least one of the two Chromosomes 4. Consequently, the heterozygote genotype of the HLA-A29-restricted UGT2B17 is not detectable. The immunogenic phenotype can be observed at high frequency in the White population (96.5%). In the Asian/Pacific, Black (70.9), and Mexican Mestizo (62.4%) populations, the frequency is significantly lower (*p <* 0.001). The frequency in the Cape Colored population is even lower (47.2%, Asian/Pacific, Black, and Mexican Mestizo versus Cape colored: *p <* 0.001). The lowest frequency is displayed by the Mulatto population (22.0%, Cape Colored versus Mulatto: *p <* 0.01).

### Linkage Analysis of the ACC-1 and ACC-2 Alleles

Linkage analyses of the ACC-1/ACC-2 haplotypes in Whites who are homozygous for ACC-1 and/or ACC-2, yield a correlation coefficient *r^2^* of 0.91 between the alleles of ACC-1 and ACC-2, indicating the existence of immunogenic and nonimmunogenic ACC-1/ACC-2 haplotypes (Table 4). In the other ethnic populations, the correlation is much weaker, and clearly absent in the Black population (*r^2^* = 0.18).

**Table 4:**
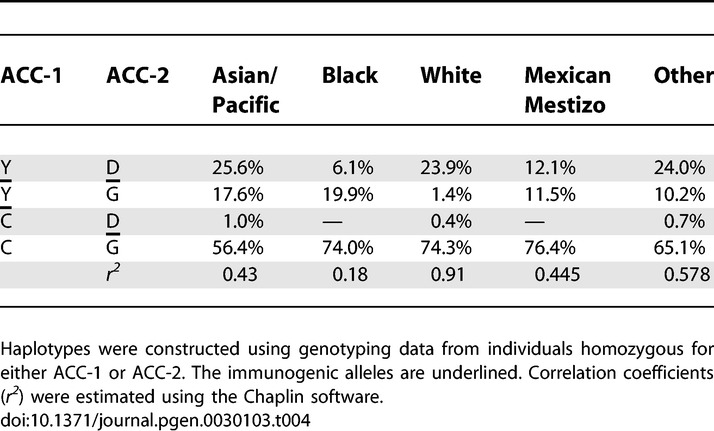
Analysis of ACC-1/ACC-2 Haplotypes

### Geospatial Analysis of White Minor H Antigen Frequencies

Due to the density of the number of geographical locations and of individuals from the identical ethnic background per location, reliable geospatial analysis could only be executed on the White population.

#### HA-1.

**Figure 1:**
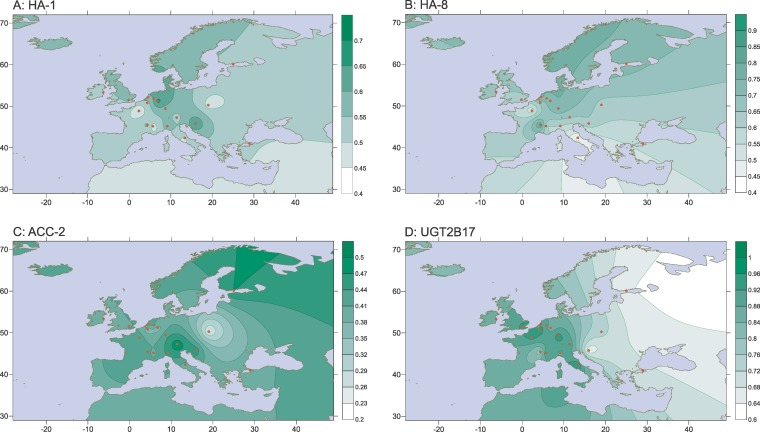
Geospatial Analyses of the Phenotype Frequencies of HA-1 (A), HA-8 (B), ACC-2 (C), and UGT2B17 (D) in the European White Populations Red dots represent the centers from which the frequency data were obtained. Numbers in the scaling indicate the proportion of individuals with an immunogenic phenotype.

Geospatial analysis was performed on the HA-1 immunogenic phenotype frequencies within the White population. As shown in Figure 1A, no correlation between the HA-1 phenotype frequency and the geographic localization can be observed within Europe.

#### HA-2.

A large geospatial diversity in HA-2 phenotype frequencies is observed among Whites grouped by location, ranging from approximately 70% positive for the immunogenic phenotype in Whites from Seattle and Baltimore to 95% in the British, Irish, Dutch, German, and Polish samples (unpublished data). However, no clear geographic pattern is observed within the European White population (Figure S1B).

#### HA-3.

The phenotype frequencies of HA-3 within the White population do not display a geographical pattern.

#### HA-8.

Geospatial analysis of the HA-8 phenotype frequencies strongly indicates the presence of a north–south gradient, with phenotype frequencies of more than 65% in The Netherlands, Germany, and Finland, approximately 60% in Central Europe, Croatia and Poland, and Northern Italy, and less than 55% in Turkey and Southern Italy (Figure 1B).

#### HB-1.

The phenotype frequencies of HB-1^H^ (Figure S1E) and HB-1^Y^ (unpublished data) are very diverse. No geographical pattern can be detected for the HB-1 minor H antigens.

#### ACC-1 and ACC-2.

Due to the strong linkage between the alleles of ACC-1 and ACC-2 in the White population (Table 4), the geospatial analyses of these two minor H antigens yield nearly identical patterns (Figure 1C and Figure S1F). The results suggest a higher frequency of the immunogenic phenotypes in the central part of Europe, but a clear geographical pattern cannot be detected.

#### SP110.

The SP110 phenotype frequencies within the White population show diversity when geospatially analyzed (Figure S1H). However, no distinct pattern can be observed.

#### PANE1.

The phenotype frequencies of PANE1 of the various White populations do not display a geographical pattern.

#### UGT2B17.

Almost all individuals within the White population express the UGT2B17 immunogenic phenotype (96.5%), while the nonimmunogenic phenotype is nearly absent (3.5%). With frequencies of 90%–100% in Western Europe and less than 65% in the eastern parts, the immunogenic phenotype clearly decreases in the eastward direction (Figure 1D). Although data from the Asian/Pacific population are limited to two subpopulations, i.e., the Asian Indian population two locations) and the South East Asian population from Taiwan and Japan, they indicate that the immunogenic UGT2B17 phenotype further decreases in the eastward direction (Asian Indian population 58.7%, South East Asian population 33.3%), suggesting the presence of a more global east–west gradient in Eurasia.

### Estimation of Minor H Antigen Disparity Rates among HLA-Matched Related and Unrelated Individuals

The information on the phenotype frequencies subsequently allows calculation of the chance of having a minor H antigen disparate donor/recipient pair in the HLA-matched related (sib) and unrelated (matched unrelated donor, MUD) SCT settings. Frequencies of HLA molecules also vary among the ethnic populations (http://www.allelefrequencies.net). Consequently, the theoretical estimations of the minor H antigen phenotypic disparities in HLA-matched pairs are corrected for the frequency of the relevant HLA restriction molecule in each ethnic population.

#### HA-1.

In the White population the estimated disparity rates for the HLA-A2-restricted minor H antigen HA-1, and thus corrected for the frequency of HLA-A2 in the White population, are 6.6% for sib pairs (Table 5) and 12.0% for MUD pairs (Table 6). Disparity rates in the Mexican Mestizo and Mulatto populations were slightly higher. Lower disparity rates are estimated for the Asian/Pacific and the Black populations, due to differences in both the frequency of the immunogenic HA-1 phenotype and the HLA-A2 molecule.

**Table 5:**
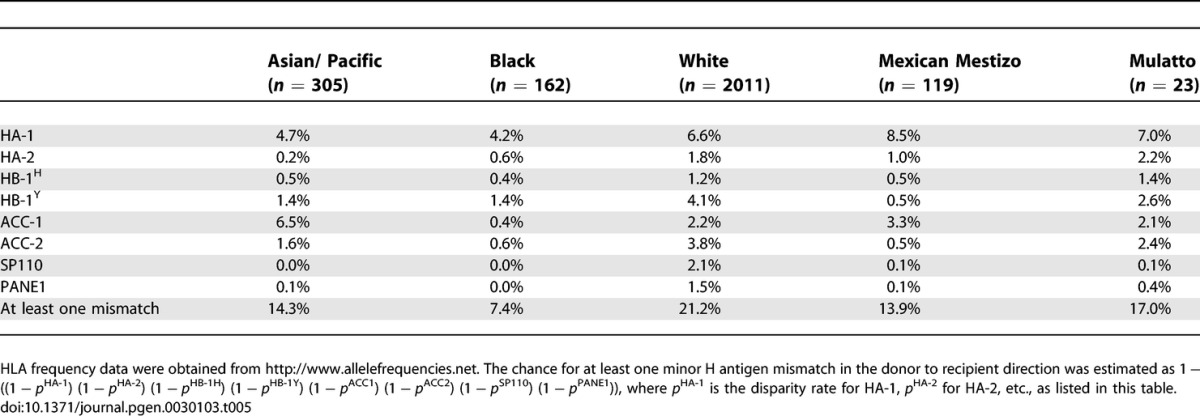
Estimated Disparity Rates for Hematopoietic Minor H Antigens in HLA-Matched Transplant Sib Pairs Corrected for the Frequencies of the HLA Restriction Molecule

**Table 6:**
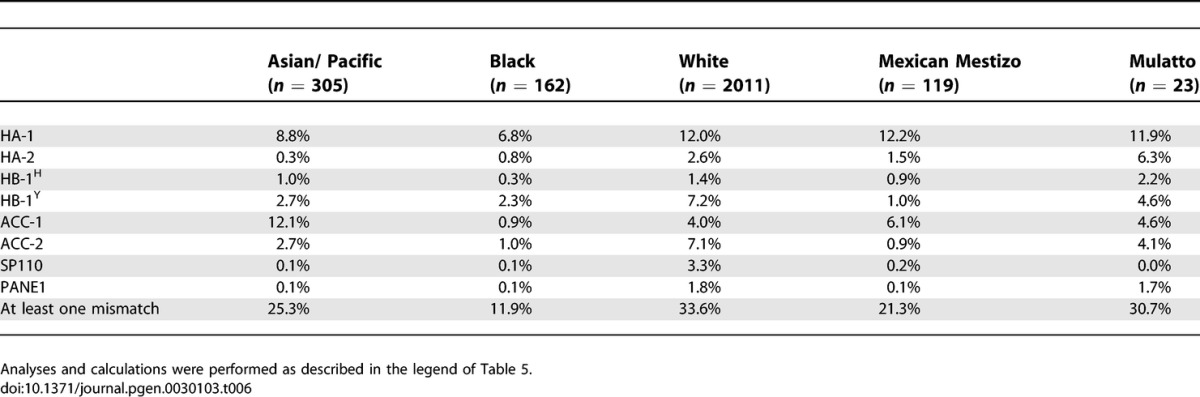
Estimated Disparity Rates for Hematopoietic Minor H Antigens in MUD Transplant Pairs Corrected for the Frequencies of the HLA Restriction Molecule

#### HA-2.

Due to the high frequency of the immunogenic HA-2 phenotype in all populations, the estimated theoretical disparity rates for HA-2, corrected for the HLA-A2 frequencies, are low (sib pairs 0.2%–2.2%, MUD pairs 0.3%–6.3%; Tables 5 and 6). In the Asian/Pacific population HA-2 disparity is nearly absent (0.2% in sib pairs and 0.3% in MUD pairs). Significant immunological disparity for HA-2 is restricted to the White (2.6%) and Mulatto (6.3%) in MUD pairs.

#### HA-3.

Based upon the frequency data of the HA-3 immunogenic phenotype and the HLA-A1 frequencies, HA-3 disparity in sib pairs is below 2% in all populations (Table 7). Highest disparity rates are estimated for MUD pairs in the Black (2.1%), the White (3.4%), and the Mexican Mestizo (2.9%) populations (Table 8).

**Table 7:**
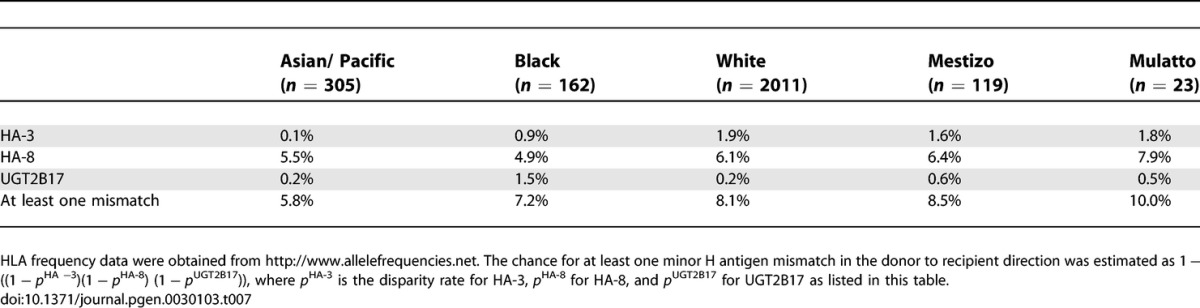
Estimated Disparity Rates for Broadly Expressed Minor H Antigens in HLA-Matched Transplant Sib Pairs Corrected for the Frequencies of the HLA Restriction Molecule

**Table 8:**
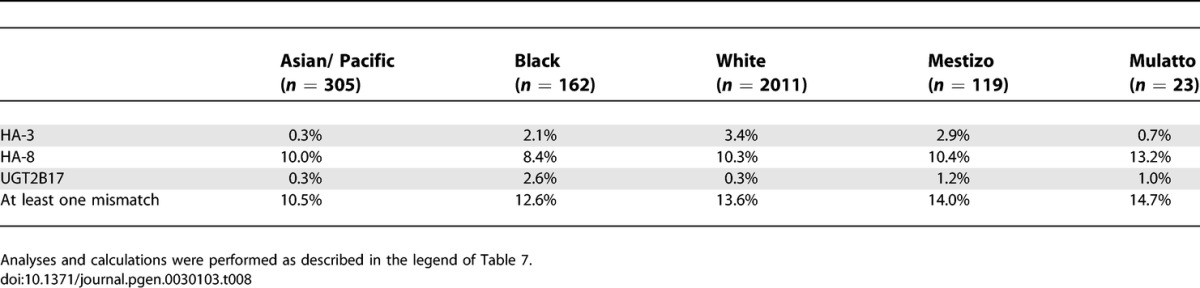
Estimated Disparity Rates for Broadly Expressed Minor H Antigens in MUD Transplant Sib Pairs Corrected for the Frequencies of the HLA Restriction Molecule

#### HA-8.

The disparity rates for the HLA-A2-restricted HA-8 are the lowest in the Black population with 4.9% in sib pairs and 8.4% in MUD pairs (Tables 7 and 8), and the highest in the Mulatto population (sib pairs: 7.9%; MUD pairs: 13.2%). All other ethnic populations display comparable disparity rates (sib pairs: 6.1%–7.9%; MUD pairs: 10.0%–10.4%).

#### HB-1.

The estimated disparity in the White and the Mulatto population for the HLA-B44-restricted HB-1^H^ is 1.2% and 1.4%, respectively, in the sib pairs and 1.4% and 2.2% in the MUD pairs, respectively. For all populations, the disparity rate for the HLA-B44-restricted HB-1^Y^ is higher than the rates for HB-1^H^ disparity (sib pairs: 0.5%–4.1%; MUD pairs: 1.0%–7.2%). The combined calculation on disparity rates for the bidirectional immunogenic HB-1 phenotypes, i.e., HB-1^H^ and the HB-1^Y^, predicts the highest HB-1 disparity in the White population (sib pairs: 5.2%; MUD pairs: 8.5%) and in the Mulatto population (sib pairs: 4.0%; MUD pairs: 6.6%).

#### ACC-1.

The estimated disparity rates for the HLA-A24-restricted ACC-1 in all populations were between 0.4%–6.5% in sib pairs and between 0.9%–12.1% for MUD pairs. This relatively broad range is caused by the highly variable frequency of HLA-A24, which is over 50% in the Asian/Pacific populations and less than 5% in the Black population.

#### ACC-2.

Disparity for the HLA-B44-restricted ACC-2 minor H antigen is low in the Black and Mexican Mestizo population (sib pairs and MUD pairs: <1.0%) Highest disparity rates were estimated for the Caucasian population (sib pairs: 3.8%; MUD pairs: 7.1%).

#### SP110.

SP110 disparity is nearly absent in the Asian/Pacific population (sib pairs: 0.0%; MUD pairs: 0.1%), the Black population (sib pairs: 0.0%; MUD pairs: 0.1%), the Mexican Mestizo population (sib pairs: 0.1%; MUD pairs: 0.2%), and in the Mulatto population (sib pairs: 0.1%; MUD pairs: 0.0%). Highest disparity was estimated for the White pairs (sib pairs: 2.1%; MUD pairs: 3.3%).

#### PANE1.

Disparity for the HLA-A3-restricted minor H antigen PANE1 above 1% has only been estimated for sib and MUD pairs of White origin (2.1% and 1.8%, respectively) and for MUD pairs of Mulatto origin (1.7%; Table 6).

#### UGT2B17.

Since the frequency of individuals homozygous for the *UGT2B17* gene deletion genotype is low in the White population and the HLA-A29 restriction molecule is infrequent in this population (7.5%), the estimated disparity rates for White sib and MUD pairs are low (sib pairs: 0.2%; MUD pairs: 0.3%). The combination of a higher frequency of homozygous *UGT2B17* gene deletion and the HLA-A29 restriction molecule (12.5%) in the Black population results in 1.5% estimated disparity in sib pairs and 2.6% in MUD pairs. In all other populations, the disparity rates are below 1.5% (Tables 7 and 8).

## Discussion

The present global study demonstrates the genotype and phenotype frequencies of all ten autosomally encoded minor H antigens identified at the initiation of the study, in 2,685 individuals of six different ethnic populations. Based on this information, the geospatial frequency distribution of the currently known minor H antigens is also described. Moreover, the phenotype frequencies provide for the first time a theoretical estimation for the relevance of ten minor H antigens for their potential use in minor H antigen–based immunotherapeutic protocols in SCT.

**Figure 2:**
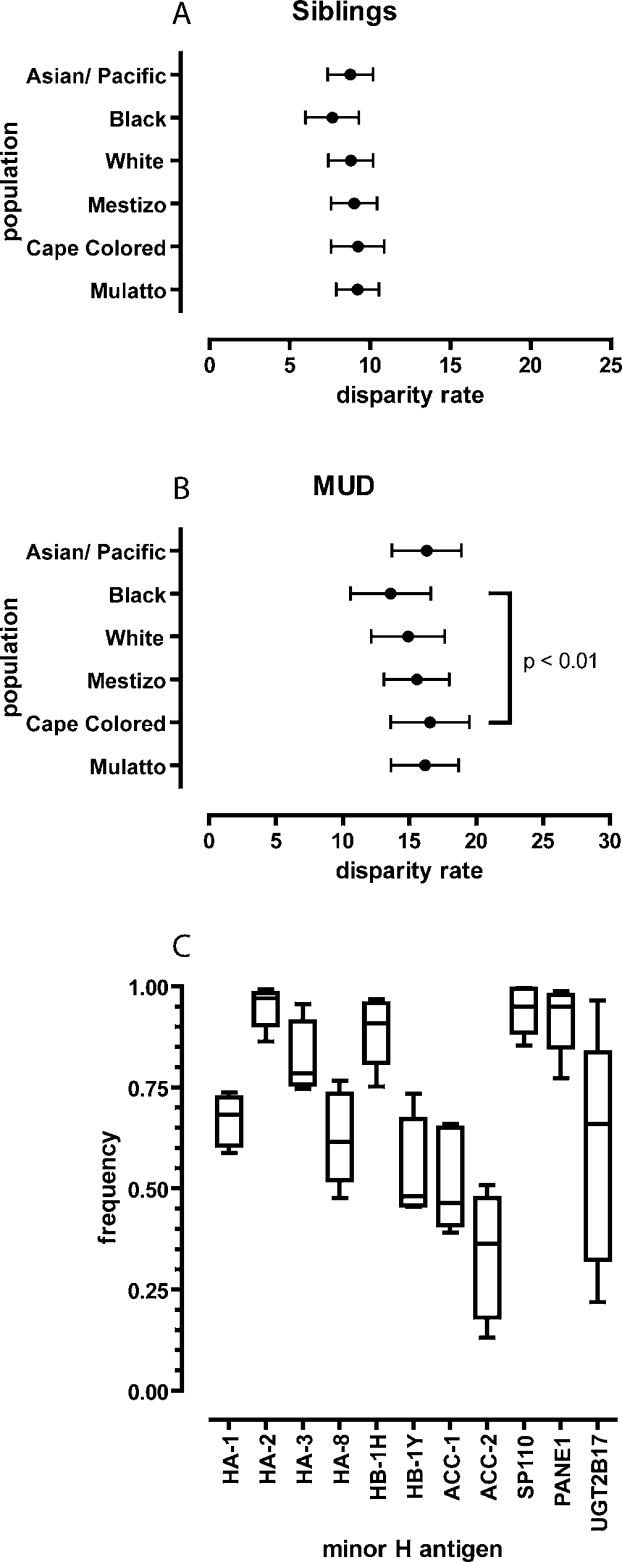
Estimated Minor H Antigen Disparity Rates Comparison for sib (A) and MUD (B) transplantations among six different ethnic populations. Data are depicted as the mean disparity rate of the ten analyzed minor H antigens and the standard error. (C) Variation in frequencies of the immunogenic minor H antigen phenotype per minor H antigen.

Small but significant differences in the genotype and phenotype frequencies in the various ethnic populations were observed for all autosomally encoded minor H antigens. The highest variation among the ethnic populations was observed for UGT2B17 (Figure 2C). The UGT2B17 frequency data in the White population are in line with previous studies [22]. As mentioned earlier, the UGT2B17 immunogenic phenotype results from homozygous deletion of the entire *UGT2B17* gene. Interestingly, homozygous *UGT2B17* gene deletion seems to be tolerated differently in the various ethnic populations.

In the present study, we also addressed the issue of linked alleles by analyzing ACC-1 and/or ACC-2 homozygous individuals. The two SNPs responsible for the ACC-1 and ACC-2 minor H antigens are both located on the *BCL2A1* gene [25]. The distance between these two SNPs is only 189 bp. Cellular recognition assays indicated that, within the White population from the Centre d'Etude du Polymorphisme Humain, the immunogenic alleles of ACC-1 and ACC-2 are linked [26]. The same study, however, also reported on Japanese individuals positive for only one of the two minor H antigens, thus providing evidence that the ACC-1 and ACC-2 are defined by distinct SNPs, which was confirmed by the characterization of the two relevant minor H peptides. In the White population, a strong linkage between the two alleles of ACC-1 and ACC-2 was indeed observed (Table 4). However, in the other ethnic populations, haplotypes containing an immunogenic allele for one and a nonimmunogenic allele for the other *BCL2A1* polymorphism occur relatively frequently. These observations have important implications for minor H antigen typing, particularly in non-White individuals, where genotyping for both the ACC-1 and the ACC-2 minor H antigen may be essential.

The availability of the large dataset on the phenotype frequencies of ten minor H antigens in the White population (2,011 White individuals from 22 different geographical locations) allowed for the performance of geospatial analyses. Geographical correlations were observed for HA-8 and UGT2B17. A distinct north–south gradient for the minor H antigen HA-8 was found, with high frequencies for the immunogenic phenotype in the northern parts of Europe and lower frequencies in the Mediterranean regions. It would be of interest to specifically analyze more samples from both the Scandinavian and the Mediterranean region to further confirm these observations. North-to-south gradients have been suggested to be a more general phenomenon for SNPs. A recent study demonstrated clustering of northern and southern European populations via SNP analyses [27]. However, since none of the other SNP-based minor H antigens demonstrated a north-to-south frequency gradient, we cannot confirm this suggestion.

The geospatial analyses of the White population on the European continent indicate a strong west-to-east correlation between the geographic location and the phenotype frequency of UGT2B17. With 58.7% in India, 32.1% in Taiwan, and 30.0% in Japan, the current Asian dataset suggests that the west-to-east gradient of UGT2B17 extends further eastwards in Eurasia. Additional data from regions such as Eastern Europe and the Middle East are required to strengthen this supposition.

Apart from HA-8 and UGT2B17, no geographical correlation could be detected for the other eight minor H antigen phenotype frequencies. A previous study analyzing geospatial distribution observed a north-to-south gradient for HA-1 within Europe [28]. The data included in the latter meta-analysis match our data and our extrapolation to Spain and Greece. Indeed, if only samples from the same regions (countries: The Netherlands, Germany, and Italy) had been analyzed using our data, a similar conclusion could have been drawn. However, we observed a large variation in the frequency of the immunogenic HA-1 phenotype in central Europe, leading to an absence of a clear north–south gradient for HA-1 in our study. The limited number of locations and the fact that these locations were oriented along a north-to-south axis, thereby skewing the results towards a similar orientation of the gradient, may have influenced the conclusions of Di Terlizzi, et al. [28].

Minor H antigens are peptides arising from genomic polymorphisms. Interestingly, most minor H antigens display a unidirectional immune recognition pattern, where the one allele is immunogenic and the other nonimmunogenic. Various mechanisms have been reported to be responsible for this phenomenon of “one allele immunogenicity.” One of these mechanisms described is via differential antigen processing of HA-3 by the proteasome [29] or by TAP translocation of HA-8 [30]. The lack of a nonimmunogenic counterpart can also have a genetic basis. These cases involve mechanisms such as gene deletion of UGT2B17 [31], frame-shift due to nucleotide insertion for LRH-1 [32], and introduction of a translation termination codon for PANE1 and ECGF1 [12,33]. As discussed above, the minor H antigen alloimmune responses are generally unidirectional. Consequently, the immune responses between minor H antigen disparate HLA-matched sibs and MUD pairs are similarly unidirectional.

The obtained information on the global phenotype frequencies of the minor H antigens known to date allows estimations on the disparity rates between putative HLA-matched stem cell donor and recipient pairs. The clinical relevance of minor H antigens currently lies in their curative effect following SCT. Minor H antigens qualifying for usage in the GvT effect of SCT are those with well-defined restricted cell and tissue expression. Earlier, dissection of minor H antigens into broadly expressed and expression restricted to the hematopoietic system has been described [7]. Interestingly, the hematopoietic minor H antigens HA-1 and ACC-1/ACC-2 show additional expression on carcinomas [9–11]. The calculations of the current clinically relevant minor H antigens are thus based on both their unidirectional immunogenicity and their hematopoietic—and in case of HA-1 and ACC-1/ACC-2, hematopoietic and solid tumor—expression profiles. The minor H antigen disparity rate calculations require additional correction of the HLA restriction molecules that are also differentially distributed among the various ethnic populations. For correct estimations of the current minor H antigen clinical potential, all of the above characteristics have been taken into account. The results can be summarized as follows. The high phenotype frequency of HLA-A2, in combination with a relatively high disparity rate for HA-1 in all ethnic populations studied, currently marks HA-1 as the most favorable minor H antigen for clinical application in all ethnic populations. Similar results, although limited to only one or two ethnic groups, were obtained for the minor H antigens ACC-1, ACC-2, and HB-1^Y^. The ACC-1 minor H antigen is restricted to the Asian/Pacific population, with 6.5% disparity in sib pairs and 12.1% in MUD pairs. Similarly, ACC-2 can only be applied in a significant proportion of the pairs in the White and Mulatto population, as counts for HB-1^Y^. Finally, estimating the chance of having at least one mismatch for a hematopoietic system–restricted minor H antigen out of the eight hematopoietic minor H antigens studied herein, revealed 21.2% and 33.6% for White sib and MUD pairs respectively, and 7.4% and 11.9% for the Black population, respectively. In all of these estimations, the HLA allele frequencies of the respective populations were taken into account.

The estimations of the minor H antigen disparity rates between HLA-matched pairs strongly differ among the various ethnic populations, raising the question of whether some populations display more phenotypic diversity than others. To address this issue, we subjected the mean genetic disparity rates per population to a paired *t-*test. The overall genetic disparity is lowest in the Black population (Figure 2). It remains unclear which mechanism forms the basis for this phenomenon. The Black population analyzed in this study comprised two major subpopulations, i.e., the African Black and the African American population. In contrast with the African Black population, the African American population has considerable Caucasoid admixture (E. D. du Toit, personal communication). We therefore performed a separate analysis of the minor H antigen phenotype frequencies in these two subpopulations for all minor H antigens. Although a tendency towards White minor H antigen phenotype frequencies was observed for the African American population, the differences didn't reach statistical significance (unpublished data). Similarly, a subanalysis of the Asian/Pacific population yielded no major regional differences except for the UGT2B17 minor H antigen (unpublished data).

The clinical potential of the current set of identified minor H antigens is clearly limited and points to enlargement of the pool of clinically relevant minor H antigens. Reverse immunology has recently been applied for identifying new minor H antigens [34]. This approach uses computer algorithms such as SYFPEITHI [35] and Bimas [36] to predict binding of polymorphic peptides to the HLA allele of choice. Accordingly, the *UGT2B17* gene could be analyzed for new minor H peptides binding to frequently occurring HLA alleles in the various ethnic populations (Table S2). Similar analyses could be executed for HB-1^H^ for its relevance in the Mexican Mestizo population, and HA-2 and PANE1 in the Brazilian Mulatto population. Finally, to enlarge the pool of minor H antigens potentially applicable in a stem cell–based protocol for solid tumors, the reverse immunology approach can be helpful in searching for minor H antigens with restricted expression on carcinoma cells [9–11]. Here, the search is specifically focused on SNPs in genes involved in tumorigenesis [5].

In conclusion, we here report differences in minor H antigen genotype and phenotype frequencies among and within the major ethnic populations. Some of these differences are geographically correlated, while others seem to be more randomly distributed. Moreover, the potential clinical application of hematopoietic specific minor H antigens as immunotherapeutic tools in SCT for hematological malignancies was estimated. The theoretical chance of having a difference for at least one hematopoietic minor H antigen in the whole study group, thus corrected for the HLA frequencies, is 11.9% to 33.6% in HLA-matched unrelated pairs.

## Materials and Methods

### Sample selection and population description.

The study population consisted of 2,685 locally available randomly selected healthy subjects and transplant donors. All research samples and data were collected according to the subjects' guidelines and protocols of the local institutional review boards. Individuals with an unknown ethnical background were excluded from the analyses.

### Minor H antigen typing.

DNA was isolated using the local standard procedures used for HLA typing. Genotyping of ten autosomally encoded minor H antigens and of HY was performed using the PCR-SSP technique (Invitrogen, http://www.invitrogen.com). The details of the minor H antigen typing methodology have been described before [37]. Previously typed cells from the Centre d'Etude du Polymorphisme Humain-Human Genome Diversity Project Cell Line Panel (CEPH panel) and from the reference DNA samples from the University of California, Los Angeles, International DNA Exchange Program were used as controls. All PCR-SSP samples were analyzed on agarose gel. If the internal control PCRs failed for one of the two allele of a particular minor H antigen, the typing for that minor H antigen was marked as incomplete and data were excluded from the analyses. The alleles of each minor H antigen were reported as the amino acid resulting from the relevant SNP. Typing data and sample parameters were submitted to a central online database (http://www.lumc.nl/ihiw14).

### Statistical analyses.

Typing data were analyzed at three levels: (1) the allele frequency, (2) genotype frequency, and (3) phenotype frequencies. Allele and genotype frequencies except those of UGT2B17 were calculated by direct counting. Since the minor H antigen UGTB17 is the result of gene deletion, the applied methodology did not allow detection of UGT2B17 heterozygosity. Therefore the UGT2B17 allele frequencies were estimated as 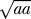 for the nonimmunogenic allele and 1 − 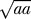 for the immunogenic allele, where “*aa*” is the frequency of the nonimmunogenic phenotype. The heterozygosity frequencies (Aa) for this minor H antigen were estimated by 2 × (1 − 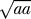 ) × (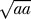 ), assuming a Hardy-Weinberg equilibrium.

Observed genotype frequencies were tested against Hardy-Weinberg equilibrium. The phenotype frequencies have been deduced from the genotype frequencies. Individuals who were homozygous or heterozygous positive for the immunogenic allele were regarded as phenotypically positive. Individuals carrying two nonimmunogenic alleles were marked as phenotypically negative. Since the HB-1 minor H antigen has been demonstrated to be immunogenic in either direction, phenotype frequencies have been calculated separately for the HB-1^H^ and the HB-1^Y^ allele as immunogenic alleles. The statistical significance of differences in allele, genotype, and phenotype frequencies between the various populations and geographic regions were determined by Chi-square analysis. The SPSS statistical software system (SPSS 11.0, http://www.spss.com) was used for these analyses. *p-*values lower than 0.05 were considered statistically significant.

Linkage of the alleles of ACC-1 and ACC-2 was analyzed with the case-control haplotype inference (Chaplin) software [34]. Individuals who were heterozygous for both ACC-1 and ACC-2 were classified as noninformative and excluded from the analysis.

### Estimations of the minor H antigen disparity rates.

The disparity rate is defined as the chance of a putative transplant recipient with a minor H antigen immunogenic phenotype having an HLA-identical related or HLA-matched unrelated stem cell donor being homozygous for the nonimmunogenic allele of the same minor H antigen encoding gene. In the case of a putative HLA-matched unrelated donor selection, the disparity rate is the frequency of homozygous immunogenic (AA) and heterozygous (Aa) individuals multiplied by the frequency of homozygous nonimmunogenic individuals (aa): ((*AA*) + (*Aa*)) × (*aa*). Sib transplantation disparity rates were estimated by 3/16(*Aa*)^2^ + 1/2(*Aa*)(*aa*). For both related and unrelated situations, the rates were multiplied by the phenotype frequency of the relevant HLA restriction molecule. To investigate the level of disparity within each population, two-tailed Wilcoxon signed rank tests were performed.

### Spatial frequency distribution analysis.

Spatial frequency distribution maps for each of the immunogenic minor H antigen phenotype frequencies were constructed for the White population samples using the Kriging procedure [38] with the Surfer 8 software (Golden Software, http://www.goldensoftware.com). For these analyses the geographical area between 29.000/−28.000 (latitude/longitude) and 72.000/49.000, covering the European continent, was split into a 1,000 × 550 squares grid using the standard software settings. Cartesian coordinates of the participating institutes were obtained from the Getty Thesaurus of Geographic Names Online (http://www.getty.edu/research/conducting_research/vocabularies/tgn).

## Supporting Information

Figure S1 Geospatial Analyses of All Minor H Antigen Phenotype Frequencies in the European White Populations Red dots represent the centers from which the frequency data were obtained. Numbers in the scaling indicate the proportion of individuals with an immunogenic phenotype.
(19.6 MB AI).

Table S1 Phenotype Data of Ten Autosomally Encoded Minor H Antigens in Six Different Ethnic Populations as Derived from the Genotyping Frequencies A “+” indicates the immunogenic phenotype and a “−” indicates the nonimmunogenic phenotype.
(68 KB DOC)

Table S2 Estimated Phenotype Disparity Rates Independent of the Frequencies of the HLA restriction molecule in sib (A) and MUD (B) transplantation settings Disparity rates were estimated as described in the Materials and Methods section using the genotype data results.
(71 KB DOC)

### Accession Numbers

The National Center for Biotechnology Information (NCBI) Gene database (http://www.ncbi.nlm.nih.gov/entrez/query.fcgi?db=gene) accession numbers for the minor H antigen genes used in the study are: *HMHA1* (HA-1), 23526; *MYO1G* (HA-2), 64005; *AKAP13* (HA-3), 11214; *KIAA0020* (HA-8), 9933; *HMHB1* (HB-1), 57824; *BCL2A1* (ACC-1 and ACC-2), 597; *SP110* (SP110), 3431; *CENPM* (PANE1), 79019; *UGT2B17* (UGT2B17), 7367.

## Abbreviations

GvHD: graft-versus-host disease
GvT: graft-versus-tumor
H: histocompatibility
HLA: human leukocyte antigen
HSCT: hematopoietic stem cell transplantation
MUD: matched unrelated donor
SCT: stem cell transplantation
