# Supplementary figures and images for: Phenotype Frequencies of Autosomal Minor Histocompatibility Antigens Display Significant Differences among Populations
Source: PLoS Genet. 2007 Jun 29;3(6):e103. doi: 10.1371/journal.pgen.0030103 (PMC1904367; doi:10.1371/journal.pgen.0030103)

A: HA-1

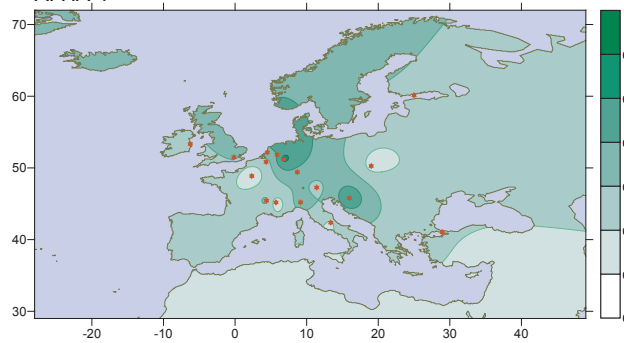

B: HA-2

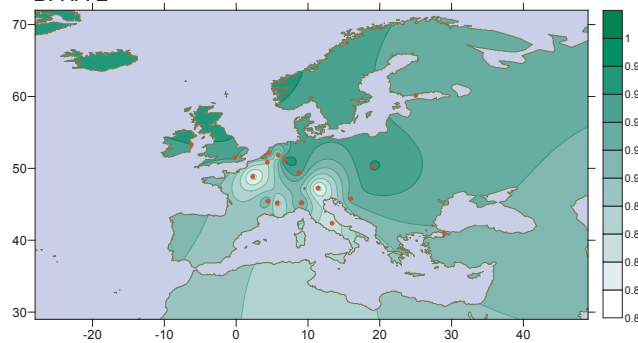

C: HA-3

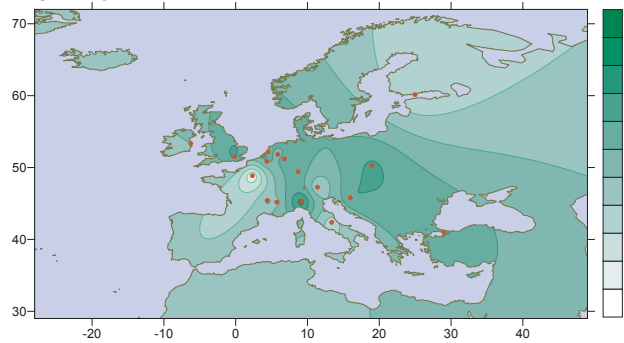

D: HA-8

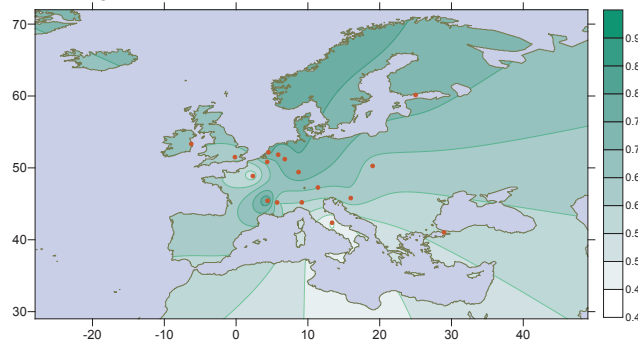

E: HB-1H

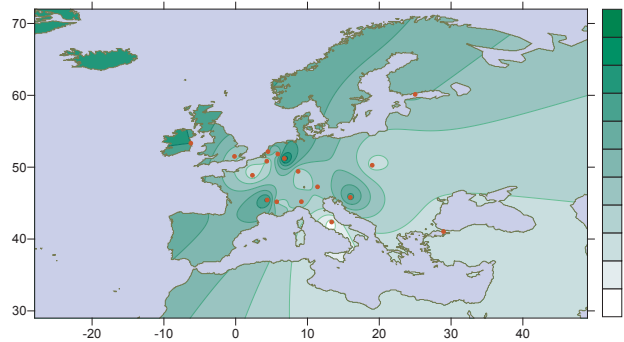

F: ACC-1

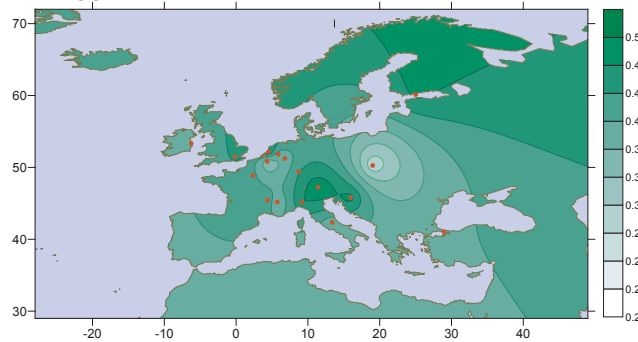

G: ACC-2

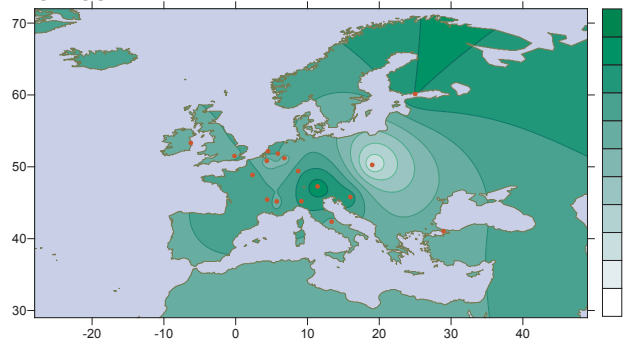

H: SP110

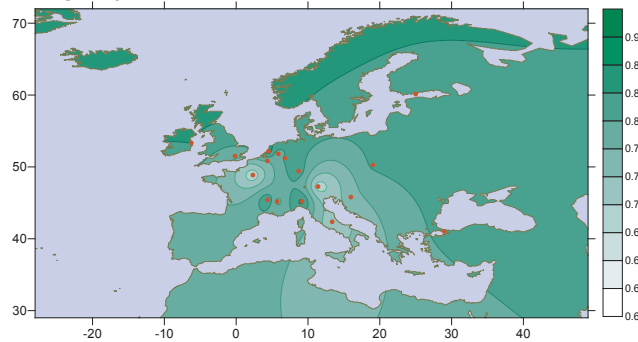

I: PANE1

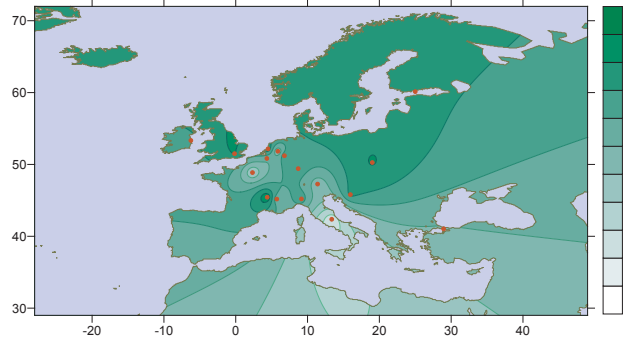

J: UGT2B17

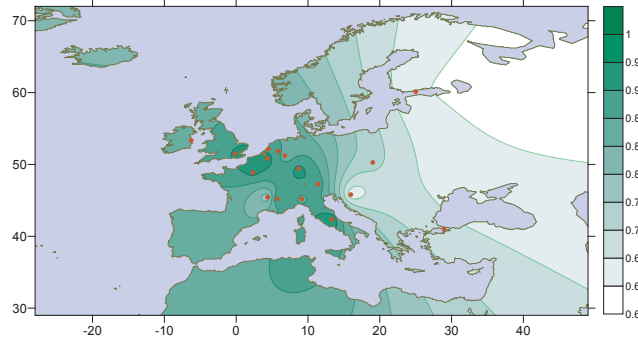

Supplement: Figure S1 — Red dots represent the centers from which the frequency data were obtained. Numbers in the scaling indicate the proportion of individuals with an immunogenic phenotype. (19.6 MB AI). [file pgen.0030103.sg001.pdf]
