# Supplementary material for: Phenotype Frequencies of Autosomal Minor Histocompatibility Antigens Display Significant Differences among Populations
Source: PLoS Genet. 2007 Jun 29;3(6):e103. doi: 10.1371/journal.pgen.0030103 (PMC1904367; doi:10.1371/journal.pgen.0030103)
Supplement: Table S1 — A “+” indicates the immunogenic phenotype and a “−” indicates the nonimmunogenic phenotype. (68 KB DOC) [file pgen.0030103.st001.doc]

**Supplementary table I**: Phenotype data of 10 autosomally encoded minor H antigens in 6 different ethnic populations as derived from the genotyping frequencies. A “+” indicates the immunogenic phenotype and a “-” indicates the non-immunogenic phenotype.

|  | | **Asian/ Pacific** | **Black** | **Caucasian** | **Mexican Mestizo** | **Cape Colored** | **Mulatto** | **Total** |
| --- | --- | --- | --- | --- | --- | --- | --- | --- |
|  |  | **(n=305)** | **(n=162)** | **(n=2011)** | **(n=119)** | **(n=65)** | **(n=23)** | **(n=2685)** |
| **HA-1** | **+** | 71.2% | 73.7% | 58.8% | 69.8% | 62.5% | 66.7% | 61.4% |
|  | **-** | 28.8% | 26.3% | 41.2% | 30.2% | 37.5% | 33.3% | 38.6% |
| **HA-2** | **+** | 99.2% | 97.5% | 94.5% | 97.3% | 96.8% | 86.4% | 95.2% |
|  | **-** | 0.8% | 2.5% | 5.5% | 2.7% | 3.2% | 13.6% | 4.8% |
| **HA-3** | **+** | 76.8% | 78.4% | 87.0% | 78.6% | 74.6% | 95.7% | 85.0% |
|  | **-** | 23.2% | 21.6% | 13.0% | 21.4% | 25.4% | 4.3% | 15.0% |
| **HA-8** | **+** | 63.2% | 59.9% | 70.2% | 76.7% | 47.6% | 56.5% | 68.5% |
|  | **-** | 36.8% | 40.1% | 29.8% | 23.3% | 52.4% | 43.5% | 31.5% |
| **HB-1H** | **+** | 89.6% | 96.8% | 94.8% | 75.2% | 92.1% | 87.0% | 93.5% |
|  | **-** | 10.4% | 3.2% | 5.2% | 24.8% | 7.9% | 13.0% | 6.5% |
| **HB-1Y** | **+** | 49.8% | 45.6% | 46.3% | 73.4% | 46.0% | 60.9% | 47.9% |
|  | **-** | 50.2% | 54.4% | 53.7% | 26.6% | 54.0% | 39.1% | 52.1% |
| **ACC-1** | **+** | 66.0% | 42.8% | 46.5% | 46.2% | 64.1% | 39.1% | 48.5% |
|  | **-** | 34.0% | 57.2% | 53.5% | 53.8% | 35.9% | 60.9% | 51.5% |
| **ACC-2** | **+** | 50.8% | 13.0% | 44.5% | 23.2% | 42.2% | 30.4% | 42.1% |
|  | **-** | 49.2% | 87.0% | 55.5% | 76.8% | 57.8% | 69.6% | 57.9% |
| **SP110** | **+** | 91.8% | 98.8% | 85.3% | 94.7% | 95.3% | 100.0% | 87.5% |
|  | **-** | 8.2% | 1.3% | 14.7% | 5.3% | 4.7% | 0.0% | 12.5% |
| **PANE1** | **+** | 93.4% | 98.8% | 92.6% | 96.7% | 96.9% | 77.3% | 93.2% |
|  | **-** | 6.6% | 1.2% | 7.4% | 3.3% | 3.1% | 22.7% | 6.8% |
| **UGT2B17** | **+** | 69.6% | 70.9% | 96.5% | 62.4% | 42.7% | 22.0% | 85.3% |
|  | **-** | 30.4% | 29.1% | 3.5% | 37.6% | 57.3% | 78.0% | 14.7% |
