# Supplementary material for: Phenotype Frequencies of Autosomal Minor Histocompatibility Antigens Display Significant Differences among Populations
Source: PLoS Genet. 2007 Jun 29;3(6):e103. doi: 10.1371/journal.pgen.0030103 (PMC1904367; doi:10.1371/journal.pgen.0030103)
Supplement: Table S2 — Disparity rates were estimated as described in the Materials and Methods section using the genotype data results. (71 KB DOC) [file pgen.0030103.st002.doc]

**Supplementary Table II**: Estimated phenotype disparity rates independent from the frequencies of the HLA restriction molecule in sibling (A) and MUD (B) transplantation settings. Disparity rates were estimated as described in the Materials and Methods section using the genotype data results.

| **A** | **Asian/ Pacific** | **Black** | **Caucasian** | **Mexican Mestizo** | **Cape Colored** | **Mulatto** | **Total** |
| --- | --- | --- | --- | --- | --- | --- | --- |
|  | **(n=305)** | **(n=162)** | **(n=2011)** | **(n=119)** | **(n=65)** | **(n=23)** | **(n=2685)** |
| **HA-1** | 11.0% | 11.9% | 13.4% | 14.7% | 15.9% | 13.0% | 13.2% |
| **HA-2** | 0.5% | 1.8% | 3.7% | 1.8% | 1.4% | 4.1% | 3.0% |
| **HA-3** | 7.3% | 7.2% | 6.4% | 9.3% | 7.6% | 10.6% | 6.8% |
| **HA-8** | 12.8% | 14.0% | 12.3% | 11.0% | 12.1% | 14.7% | 12.4% |
| **HB-1^H^** | 5.0% | 4.0% | 4.2% | 10.5% | 4.2% | 7.4% | 4.6% |
| **HB-1^Y^** | 12.8% | 14.9% | 14.2% | 10.9% | 13.0% | 13.6% | 14.0% |
| **ACC-1** | 12.1% | 11.6% | 13.5% | 13.5% | 15.4% | 11.0% | 13.4% |
| **ACC-2** | 14.4% | 6.0% | 13.3% | 9.9% | 12.8% | 12.3% | 13.0% |
| **SP110** | 3.2% | 0.5% | 7.9% | 2.3% | 4.3% | 1.3% | 6.3% |
| **PANE1** | 5.2% | 0.2% | 5.5% | 2.0% | 1.7% | 4.5% | 4.6% |
| **UGT2B17** | 12.1% | 11.9% | 2.3% | 13.1% | 13.1% | 8.8% | 7.7% |

| **B** | **Asian/ Pacific** | **Black** | **Caucasian** | **Mexican Mestizo** | **Cape Colored** | **Mulatto** | **Total** |
| --- | --- | --- | --- | --- | --- | --- | --- |
|  | **(n=305)** | **(n=162)** | **(n=2011)** | **(n=119)** | **(n=65)** | **(n=23)** | **(n=2685)** |
| **HA-1** | 20.5% | 19.4% | 24.2% | 21.1% | 23.4% | 22.2% | 23.7% |
| **HA-2** | 0.8% | 2.4% | 5.2% | 2.6% | 3.1% | 11.8% | 4.6% |
| **HA-3** | 17.8% | 16.9% | 11.3% | 16.8% | 18.9% | 4.2% | 12.8% |
| **HA-8** | 23.3% | 24.0% | 20.9% | 17.9% | 24.9% | 24.6% | 21.6% |
| **HB-1^H^** | 9.3% | 3.1% | 4.9% | 18.6% | 7.3% | 11.3% | 6.1% |
| **HB-1^Y^** | 25.0% | 24.8% | 24.9% | 19.5% | 24.8% | 23.8% | 25.0% |
| **ACC-1** | 22.4% | 24.5% | 24.9% | 24.9% | 23.0% | 23.8% | 25.0% |
| **ACC-2** | 25.0% | 11.3% | 24.7% | 17.8% | 24.4% | 21.2% | 24.4% |
| **SP110** | 7.5% | 1.2% | 12.5% | 5.0% | 4.5% | 0.0% | 10.9% |
| **PANE1** | 6.2% | 1.2% | 6.9% | 3.2% | 3.0% | 17.6% | 6.3% |
| **UGT2B17** | 21.2% | 20.6% | 3.4% | 23.5% | 24.5% | 17.1% | 12.6% |
